# Resveratrol Attenuates High Glucose-Induced Vascular Endothelial Cell Injury by Activating the E2F3 Pathway

**DOI:** 10.1155/2020/6173618

**Published:** 2020-04-28

**Authors:** Xiaolian Ding, Wei Yao, Jie Zhu, Kaida Mu, Jing Zhang, Jin-an Zhang

**Affiliations:** ^1^Department of Nephrology and Endocrinology, Weinan Central Hospital of Weinan, Shaanxi Province, China 714000; ^2^Department of Endocrinology, Shanghai University of Medicine & Health Sciences, Affiliated Zhoupu Hospital, No. 1500 Zhouyuan Road, Pudong New District, Shanghai, China 201318

## Abstract

Type 2 diabetes mellitus (T2DM) is the most common metabolic disease. High glucose-induced macrovascular disease and microangiopathy are major complications of diabetes. E2F3, a member of the E2F transcription factor family, is closely related to cardiovascular diseases. Resveratrol, a nonflavonoid polyphenolic compound widely found in plants, has been shown to have cardiovascular protection. However, there are few studies on whether resveratrol can effectively treat diabetic angiopathy, and the specific mechanism involved needs further study. This study investigated whether E2F3 transcription factors are involved in the process of vascular endothelial injury induced by high glucose and observed its effects on the proliferation of vascular endothelial cells. Then, it analyzed whether resveratrol can inhibit high glucose-induced vascular endothelial cell injury by regulating the E2F3 pathway. We demonstrated that the expression level of the E2F3 transcription factor was significantly inhibited in high glucose state. Resveratrol inhibited high glucose-induced vascular endothelial cell injury by upregulating the E2F3 pathway. High glucose can induce vascular endothelial injury by inhibiting E2F3 gene expression, while resveratrol can inhibit high glucose-induced vascular endothelial injury by activating the E2F3 pathway.

## 1. Introduction

Type 2 diabetes mellitus (T2DM), the most common metabolic disease, is mainly caused by islet *β*-cell dysfunction and insulin resistance [1]. With the improvement of living standards and lifestyle changes, the prevalence of T2DM has been increasing year by year and has become a worldwide epidemic disease, and there are currently more than 400 million T2DM patients worldwide [1]. In addition, T2DM occurs in a younger population, and more and more adolescents are beginning to suffer from T2DM [2]. Macrovascular diseases and microvascular diseases are the main complications of diabetes, and the primary disabling and lethal factors have become the core problem of diabetes treatment. Although the current hypoglycemic drugs and methods for diabetes have made great progress, these treatments are not effective in reducing the risk of diabetic vascular complications [3]. In addition to active hypoglycemic and lipid-lowering and blood pressure control, there is currently no drug that can effectively prevent or treat diabetic complications.

It has been clarified that vascular endothelial cell injury caused by high glucose is the initiating factor and the main pathogenesis of diabetic vascular disease and plays a key role in the occurrence and development of diabetic vascular complications. Excessive apoptosis and dysfunction of vascular endothelial cells caused by high glucose can increase the permeability of endothelium, thereby promoting the invasion of blood lipid components and immune cells such as monocytes into the vascular endothelium, and gradually aggravating diabetes. At present, it has been found that hyperglycemia can damage the function of vascular endothelial cells through various mechanisms, such as inflammation and oxidative stress; however, the specific molecular mechanism remains to be further studied [1]. In-depth study of the pathogenesis of vascular endothelial dysfunction induced by high glucose can help to explain the mechanism of diabetic vascular disease, and it has great clinical significance for the prevention and treatment of diabetic vascular complications.

The E2F family is a group of transcription factors closely related to cell proliferation and differentiation. It plays a key role in the regulation of cell cycle and cell proliferation and is composed of eight different members (E2F1-E2F8) [4–7]. Although E2F transcription factors are mainly involved in cell cycle regulation, each member has diverse and complex biological functions and also functions to regulate immune response and substance metabolism [8, 9]. As a member of the E2F transcription factor family, E2F3 plays an important role in regulating the cell cycle [10]. Several studies have also shown that E2F3 is closely related to cardiovascular diseases [11–13]. E2F3 knockout mice have dysplasia or congestive heart failure, suggesting that E2F3 plays an important role in the development of the cardiovascular system or in maintaining normal functions [14, 15]. In addition, E2F3 can also regulate islet *β* cell proliferation and stem cell differentiation [16, 17], indicating that E2F3 can play different regulatory functions by targeting different genes in different tissue cells [10]. At present, the role and mechanism of E2F3 in regulating vascular endothelial cell function is still unclear, and its role in the induction of vascular endothelial injury by high glucose remains unclear. Resveratrol is a nonflavonoid polyphenolic compound widely found in wine and various traditional Chinese medicines. It has significant antioxidation, anti-inflammatory, and antiaging effects. Many studies have shown that resveratrol has cardiovascular protection [18, 19]. Resveratrol can protect the cardiovascular system by improving lipid metabolism, acting against oxidation, scavenging free radicals, and inhibiting various inflammatory factors [18, 19]. However, there are few studies on whether resveratrol can effectively treat diabetic angiopathy, and the specific mechanism involved needs further study. In summary, in order to further study the pathogenesis of diabetic angiopathy, we propose the following hypotheses: E2F3 transcription factor plays an important role in regulating vascular endothelial cell function. High glucose can induce vascular endothelial injury by inhibiting the E2F3 pathway, and resveratrol can inhibit high glucose-induced vascular endothelial injury by activating the E2F3 pathway.

## 2. Materials and Methods

### 2.1. Construction of High Glucose-Induced HUVEC Injury Model

Primary human umbilical vein endothelial cells (HUVECs) were purchased (ATCC) and cultured in ECM cell culture medium at 37°C under 5% CO_2_. The medium was changed every other day, and cells of passage 3-5 were used for the experiment. When HUVECs were grown to 70-80% density, the cultured cells were randomly divided into normal control group (5.5 mmol/L glucose), high-glucose group (25.6 mmol/L glucose), and high-glucose group (33.3 mmol/L glucose). The morphological changes of cells were observed by light microscopy at 0 h, 24 h, and 48 h. The proliferation of HUVEC cells was detected by CCK-8 method. Flow cytometry Annexin V was used to detect apoptosis levels.

### 2.2. Targeted Regulation of E2F3 Expression Levels

HUVECs were randomly divided into normal control group (5.5 mmol/L glucose), high-glucose control group 2 (33.3 mmol/L glucose), high-glucose/E2F3 overexpression plasmid (33.3 mmol/L glucose + E2F3 overexpression plasmid transfection), and high-glucose/E2F3 siRNA plasmid group (33.3 mmol/L glucose + E2F3 siRNA plasmid transfection) when growing to 70-80% confluence. The E2F3 expression intervention group was preincubated with the corresponding E2F3 overexpression plasmid or E2F3 siRNA plasmid for 24 h, and the other groups were cultured under normal conditions for 24 h under the same conditions. The target sequence of the E2F3 gene for the construction of lentivirus and siRNA was 5′-UAACCUUUGAUUCUCUGAAUCCUCG-3′.

### 2.3. Resveratrol Intervention

The cells were randomly divided into blank control group, resveratrol low-concentration intervention group (10 *μ*mol/L resveratrol), and resveratrol medium-concentration intervention group (33 *μ*mol/L resveratrol), resveratrol high-concentration intervention group (100 *μ*mol/L resveratrol) and resveratrol high-concentration + E2F3 siRNA plasmid intervention group (100 *μ*mol/L resveratrol + E2F3 siRNA plasmid transfection). Cells were incubated for 24 h using the corresponding preformulated media as described above. Then the original medium was aspirated and high-glucose medium (33.3 mmol/L glucose) was added. The morphology and proliferation of HUVEC cells were detected by high-glucose stimulation of HUVEC cells for 48 h.

### 2.4. Real-Time PCR

RNA was extracted using the RNeasy kit (Qiagen), and then complementary DNA (cDNA) was synthesized from 1 mg of RNA. qRT-PCR was performed in triplicate using SYBR Green PCR Master Mix with a total reaction volume of 15 *μ*L. The reaction was carried out using an Applied Biosystems 7500 real-time PCR system. The PCR procedure was as follows: one cycle was performed at 95°C for 30 seconds, followed by 40 two-step cycles of 5 seconds at 95°C, and 34 seconds at 63°C. E2F3 upstream primer sequence: 5′-ACGTCTCTTGGTCTGCTCAC-3′, downstream primer sequence: 5′-TCTTAATGAGGTGGATGCCT-3′.

### 2.5. CCK-8

Cell suspension of 100 *μ*L was placed in a 96-well plate. The plate was preincubated in an incubator for 24 h (37°C, 5% CO _2_). We added 10 *μ*L of different concentrations of the test substance to the plate and incubated the plate in the incubator for a suitable period of time (for example, 6, 12, 24, or 48 hours). Then, 10 *μ*L of CCK8 solution was added to each well (be careful not to create bubbles in the wells that will affect the OD reading). The plate was incubated in an incubator for 1-4 hours, and then the absorbance at 450 nm was measured with a microplate reader.

### 2.6. Flow Cytometry for Apoptosis Assay

The normal cultured and induced apoptosis cells (0.5~1 × 106) were washed twice with PBS, 100 *μ*L of staining buffer and FITC-labeled Annexin V (20 *μ*g/mL) of 10 *μ*L were added, and the cells were protected from light for 30 min at room temperature. We added PI (50 *μ*g/mL) 5 *μ*L, avoiding the light reaction for 5 min, added 400 *μ*L of binding buffer, and immediately used FACS for quantitative detection by flow cytometry (not more than 1 h), without adding Annexin V-FITC and one tube of PI served as a negative control.

### 2.7. Statistical Analysis

Data are expressed as mean ± SD. A paired *t* test was used to compare E2F3 gene expression levels among the groups. *P* < 0.05 was considered statistically significant. Statistical analysis was performed using SPSS Statistics version 20 (IBM, Chicago, IL, USA). Flow cytometry data was analyzed using the FlowJo software v10.0 (Tree Star Inc, Ashland, OR).

## 3. Results

### 3.1. Changes of Cell Morphology and Proliferation Activity in High Glucose-Induced HUVEC Injury and Resveratrol Treatment

**Figure 1:**
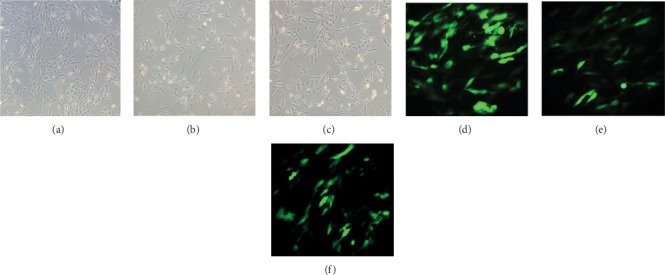
The cell morphology was significantly impaired under high glucose and partly restored by resveratrol. (a) The cell morphology in normal glucose. (b) The cell morphology in 33.3 mmol/L glucose. (c) The cell morphology in 33.3 mmol/L glucose and 100 *μ*mol/L resveratrol. (d) Transferred to E2F3 overexpressing lentivirus at normal sugar concentration. (e) Transferred to E2F3 overexpressing lentivirus at 33.3 mmol/L glucose sugar concentration. (f) Transferred to E2F3 overexpressing lentivirus at 33.3 mmol/L glucose sugar concentration and 100 *μ*mol/L resveratrol.

**Figure 2:**
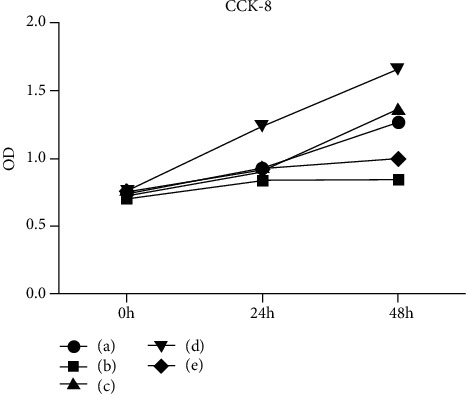
Cell proliferation activity decreased after high-glucose damage, but resveratrol could partially restore it. (a) Cell proliferation activity in normal glucose level. (b) Cell proliferation activity in 33.3 mmol/L glucose. (c) Cell proliferation activity in 33.3 mmol/L glucose and 100 *μ*mol/L resveratrol. (d) Transferred to E2F3 overexpressing lentivirus at 33.3 mmol/L glucose and 100 *μ*mol/L resveratrol. (e) Transferred to E2F3 siRNA at 33.3 mmol/L glucose sugar concentration and 100 *μ*mol/L resveratrol.

We examined the cell morphology and proliferation at different sugar concentrations and found that the cell morphology was significantly impaired under high-glucose conditions (Figure 1). The CCK8 experiment showed that high glucose inhibited cell proliferation (Figure 2). When the glucose concentration was 33.3 mmol/L, the cell proliferation curve was significantly lower than that of 5.5 mmol/L, which indicated that high-sugar concentration would inhibit the cells' proliferation activity (*P* = 0.02 at 48 h). But when 100 *μ*mol/L of resveratrol was added to the 33.3 mmol/L glucose medium, the cells' proliferation activity was restored again (*P* = 0.008 in 48 h). With a glucose concentration of 33.3 mmol/L and 100 *μ*mol/L resveratrol and transfection with E2F3 overexpression lentivirus, the cell's proliferation curve was the most significantly high, which indicated that the cells grew best. In contrast, when the cells were transfected with E2F3 siRNA, their proliferation curve was the lowest, indicating that the cells had the worst growth and suffered significant damage (*P* = 0.001 at 48 h).

### 3.2. Changes of E2F3 Expression Level in High Glucose-Induced HUVEC Injury and Resveratrol Treatment

**Figure 3:**
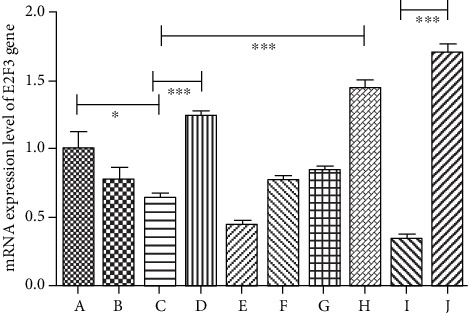
mRNA expression level of E2F3 gene. (a) 5.5 mmol/L glucose. (b) 25.6 mmol/L glucose. (c) 33.3 mmol/L glucose. (d) 33.3 mmol/L glucose + E2F3 overexpression plasmid transfection. (e) 33.3 mmol/L glucose + E2F3 siRNA plasmid transfection. (f) 33.3 mmol/L glucose + 10 *μ*mol/L resveratrol. (g) 33.3 mmol/L glucose + 33 *μ*mol/L resveratrol. (h) 33.3 mmol/L glucose + 100 *μ*mol/L resveratrol. (i) 33.3 mmol/L glucose + 100 *μ*mol/L resveratrol + E2F3 siRNA plasmid transfection. (j) 33.3 mmol/L glucose + 100 *μ*mol/L resveratrol + E2F3 overexpressing lentivirus. (∗*P* < 0.05, ∗∗*P* < 0.01, ∗∗∗*P* < 0.005).

Through real-time PCR experiments, we found that there was no significant difference in E2F3 gene expression between 5.5 mmol/L glucose and 25.6 mmol/L glucose (*P* < 0.05). But when the sugar concentration was as high as 33.3 mmol/L, E2F3 gene expression decreased significantly (*P* = 0.01). When the cells were transfected with E2F3 overexpressing lentivirus at a sugar concentration of 33.3 mmol/L, E2F3 gene expression was significantly increased (*P* = 0.0005). In contrast, when E2F3 siRNA was used, the E2F3 gene expression was significantly suppressed (*P* = 0.0004). This indicated that our plasmid transfection experiments and lentiviral transfection experiments were very successful. We further found that when resveratrol was used at high sugar levels, the expression of E2F3 increased gradually with the concentration of resveratrol, especially at 100 *μ*mol/L of resveratrol (*P* = 0.001). At high sugar concentrations, when high concentrations of resveratrol and E2F3 siRNA plasmid were used simultaneously, the expression of E2F3 was significantly suppressed. With high concentrations of resveratrol and E2F3 overexpressing lentivirus, the expression of E2F3 was the highest among all the groups. The difference between these two groups was very significant (*P* < 0.0001) (Figure 3).

### 3.3. Changes of Apoptosis Rate in High Glucose-Induced HUVEC Injury and Resveratrol Treatment

**Figure 4:**
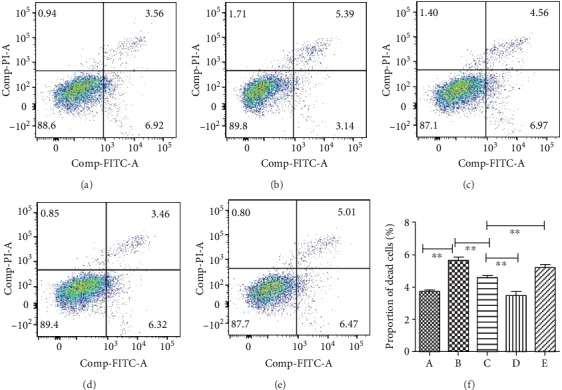
Resveratrol could reduce apoptosis induced by high glucose. (a) Apoptosis rate of cells in normal glucose. (b) Apoptosis rate of cells in 33.3 mmol/L glucose. (c) Apoptosis rate of cells in 33.3 mmol/L glucose and 100 *μ*mol/L resveratrol. (d) Apoptosis rate of cells in 33.3 mmol/L glucose and 100 *μ*mol/L resveratrol with E2F3 overexpressing lentivirus. (e) Apoptosis rate of cells in 33.3 mmol/L glucose and 100 *μ*mol/L resveratrol with E2F3 siRNA. (f) Flow cytology apoptosis rate statistical histogram. (∗*P* < 0.05, ∗∗*P* < 0.01, ∗∗∗*P* < 0.005).

Flow cytometry experiments showed that the proportion of apoptosis increased under high-glucose conditions (Figure 4). When the glucose concentration was normal, the cells had an apoptosis rate of 3.6 ± 0.32%, and when the glucose concentration was 33.3 mmol/L, the apoptosis rate of the cells increased significantly, reaching 5.6 ± 0.43% (mean ± SD) (*P* = 0.001). Importantly, we discovered that when 100 *μ*mol/L of resveratrol was added, compared with no resveratrol, the cell apoptosis rate was significantly reduced to be only 4.65 ± 0.32% (*P* = 0.002). When the E2F3 gene was overexpressed, the apoptosis rate was even lower, only 3.47 ± 0.34% (*P* = 0.002). Then we knocked down the E2F3 gene using siRNA and found that the protective effect of resveratrol was significantly weakened, and the apoptosis rate increased to 5.03 ± 0.21% (*P* = 0.006) (Figure 4).

## 4. Discussion

At present, the role and mechanism of the E2F-like transcription factor pathway in high glucose-induced vascular endothelial dysfunction remains unclear. Our study found for the first time that E2F3 was inhibited in high-glucose states, suggesting that the E2F3 transcription factor pathway may play an important role in diabetic angiopathy. Resveratrol, a widely versatile drug, was first discovered to protect vascular endothelial cells under high-glucose damage by activating the E2F3 pathway. This demonstrates the potential use of resveratrol in the treatment of diabetes.

The E2F family is a group of transcription factors closely related to cell proliferation and differentiation. It plays a key role in the regulation of cell cycle and cell proliferation and is composed of eight different members (E2F1-E2F8). [4–7]. Although E2F transcription factors are mainly involved in cell cycle regulation, each member has diverse and complex biological functions, and also functions to regulate immune response and substance metabolism [8, 9]. Many studies have demonstrated that E2F-like transcription factors play important regulatory roles in the cardiovascular system, and their dysfunction may be associated with multiple cardiovascular diseases [11, 12, 20–27]. In addition, different E2F transcription factors have significant effects on the cardiovascular system, such as different regulation of proliferation or apoptosis of vascular smooth muscle cells or cardiomyocytes [13, 28–30]. E2F3 mainly consists of two subtypes, E2F3a and E2F3b. They are encoded by a single locus through the use of different promoters and 5′ coding exons. E2F3a has been extensively characterized and is classified as a member of the activated E2F group, which includes E2F1, E2F2, and E2F3a. E2F1 is the first E2F transcription factor discovered, and there are many studies on its role in cardiovascular diseases [22, 27, 31, 32]. E2F1 is closely related to apoptosis of vascular endothelial cells, and overexpression of E2F1 can inhibit the apoptosis-promoting effect of tumor necrosis factor alpha [27]. E2F1 is also associated with abnormal proliferation of vascular smooth muscle cells caused by high glucose [32]. In addition, E2F1 can affect the formation of new blood vessels and functional recovery after myocardial infarction by downregulating the expression level of VEGF molecules [22]. Similar to E2F1, E2F2 is closely related to the function of vascular endothelial cells and cardiomyocytes, and its dysfunction may also be involved in the pathogenesis of various cardiovascular diseases [13, 26, 30]. Several studies have also shown that E2F3 is closely related to cardiovascular diseases [11–13]. E2F3 knockout mice have dysplasia or congestive heart failure, indicating that E2F3 plays an important role in the development of the cardiovascular system or maintenance of its normal functions [14, 15]. In addition, E2F3 can also regulate islet *β* cell proliferation and stem cell differentiation [16, 17].

Resveratrol, with the chemical name of 3, 4′, 5-trihydroxystilbene, is a nonflavonoid polyphenolic compound and is produced by plants (mainly seed plants) in the face of adverse conditions such as fungal infections and ultraviolet radiation. It is essentially a plant defensin that protects plants themselves and is found in more than 70 plants, such as the grape family, the lily family, and the legume family, especially abundant in grapes [33]. With the deep research on resveratrol, it has been found to have a wide range of pharmacological effects, such as antitumor, anticardiovascular disease, anti-inflammatory, immunomodulatory, antibacterial, antiviral, antiaging, and estrogen-like activities. In addition, it can alleviate the damage of tissues and organs caused by many factors and protect liver cells [18, 19]. In terms of the regulation of blood vessels, resveratrol has been shown to have a broad relaxing effect on blood vessels. Resveratrol inhibits contractile reactivity of isolated intact rat arteries to norepinephrine in a dose-dependent manner. In addition, resveratrol has also been found to be resistant to atherosclerosis and has a role in the prevention and treatment of coronary atherosclerotic heart disease. Resveratrol has even been found to reduce myocardial ischemia-reperfusion injury [34, 35]. However, we first found that the E2F3 gene of vascular endothelial cells was inhibited in the high-glucose state, which would lead to cell damage, and activation of the E2F3 pathway could protect vascular endothelial cells.

In fact, the study on resveratrol for diabetes has been widely studied in animal models and diabetic patients, but mainly focused on lowering blood sugar levels and insulin resistance. Scientists have demonstrated that resveratrol improves glucose homeostasis, reduces insulin resistance, protects islet beta cells, improves insulin secretion, and improves metabolic disorders [36, 37]. The resveratrol-induced effect is closely related to the ability of the compound to increase the expression/activity of AMPK and SIRT1 in various tissues of diabetic subjects. Furthermore, it has been shown that the antioxidant and anti-inflammatory effects of resveratrol are also seen in diabetic animals. However, the effect of resveratrol on vascular endothelium in diabetes was rarely studied. Our study demonstrates for the first time that resveratrol protects vascular endothelial cells under high-glucose induced-damage by activating the E2F3 pathway. This is very meaningful for expanding people's perception of the role of resveratrol.

In order to further study the pathogenesis of diabetic angiopathy, we have demonstrated through this experiment that the E2F3 transcription factor plays an important role in regulating vascular endothelial cell function. High glucose can induce vascular endothelial injury by inhibiting the E2F3 pathway, while resveratrol can inhibit high glucose-induced vascular endothelial injury by activating the E2F3 pathway. Interventional treatment mechanism of resveratrol on high glucose-induced vascular endothelial injury may also provide new ideas and therapeutic targets for the prevention and treatment of diabetes-related vascular lesions.

## 5. Conclusions

In conclusion, we find that high glucose can induce vascular endothelial injury by inhibiting E2F3 gene expression, while resveratrol can inhibit high glucose-induced vascular endothelial injury by activating the E2F3 pathway.

## Data Availability

The datasets used and/or analyzed during the current study are available from the corresponding author on reasonable request.

## Abbreviations

T2DM: Type 2 diabetes mellitus
HUVEC: Human umbilical vein endothelial cells
cDNA: Complementary DNA.
